# Simple Scalable Multimodal Semantic Segmentation Model

**DOI:** 10.3390/s24020699

**Published:** 2024-01-22

**Authors:** Yuchang Zhu, Nanfeng Xiao

**Affiliations:** School of Computer Science & Engineering, South China University of Technology, Guangzhou 510006, China; xiaonf@scut.edu.cn

**Keywords:** autonomous driving, visual perception, multimodal, semantic segmentation, multimodal semantic segmentation

## Abstract

Visual perception is a crucial component of autonomous driving systems. Traditional approaches for autonomous driving visual perception often rely on single-modal methods, and semantic segmentation tasks are accomplished by inputting RGB images. However, for semantic segmentation tasks in autonomous driving visual perception, a more effective strategy involves leveraging multiple modalities, which is because different sensors of the autonomous driving system bring diverse information, and the complementary features among different modalities enhance the robustness of the semantic segmentation modal. Contrary to the intuitive belief that more modalities lead to better accuracy, our research reveals that adding modalities to traditional semantic segmentation models can sometimes decrease precision. Inspired by the residual thinking concept, we propose a multimodal visual perception model which is capable of maintaining or even improving accuracy with the addition of any modality. Our approach is straightforward, using RGB as the main branch and employing the same feature extraction backbone for other modal branches. The modals score module (MSM) evaluates channel and spatial scores of all modality features, measuring their importance for overall semantic segmentation. Subsequently, the modal branches provide additional features to the RGB main branch through the features complementary module (FCM). Leveraging the residual thinking concept further enhances the feature extraction capabilities of all the branches. Through extensive experiments, we derived several conclusions. The integration of certain modalities into traditional semantic segmentation models tends to result in a decline in segmentation accuracy. In contrast, our proposed simple and scalable multimodal model demonstrates the ability to maintain segmentation precision when accommodating any additional modality. Moreover, our approach surpasses some state-of-the-art multimodal semantic segmentation models. Additionally, we conducted ablation experiments on the proposed model, confirming that the application of the proposed MSM, FCM, and the incorporation of residual thinking contribute significantly to the enhancement of the model.

## 1. Introduction

With rapid advancement of autonomous driving technology, visual perception has become an indispensable component of autonomous driving systems. In order to enable swift and safe operations by the autonomous driving decision-making system, a profound and accurate understanding of the surrounding environment is essential. While the traditional RGB cameras play a foundational role in the visual perception, the limited information acquisition has led to the integration of a growing number of sensors in autonomous driving vehicles to compensate for the limitations in the visual perception.

Recent research in the field of semantic segmentation has achieved significant progress, with some studies proposing efficient semantic segmentation methods such as [1,2,3]. These methods demonstrate remarkable results in terms of both accuracy and speed. The authors of [4] have developed a real-time semantic segmentation framework that allows researchers to easily add or replace encoders and decoders via a flexible encoding–decoding framework, providing a powerful tool for achieving efficient real-time semantic segmentation. In addition to RGB data, RGB-D data provides rich information for semantic segmentation. Numerous studies, such as [5,6,7,8,9], focus on developing more precise RGB-D semantic segmentation methods that combine depth information for more reliable segmentation results. Researchers have also explored the combination with other sensors, such as combining infrared sensors for RGB-T semantic segmentation [10,11], or integrating event cameras [12,13] for multimodal semantic segmentation. These studies not only expand the application scope of semantic segmentation, but also enhance the segmentation performance in various environments and scenarios. Cross-modal fusion is also a current research hotspot, as seen in studies like [14,15,16], which explore how to effectively integrate data from different modalities for more comprehensive semantic segmentation.

**Figure 1:**
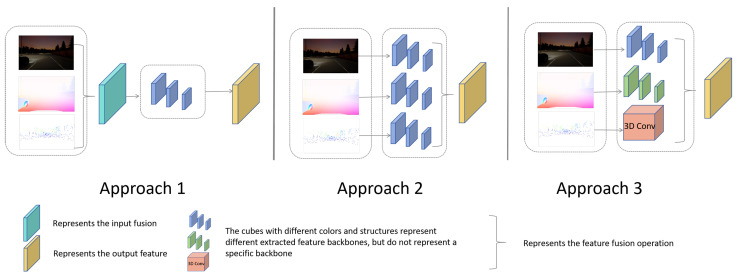
Three Approaches to Implementing Multimodal Semantic Segmentation.

There are generally three approaches to implementing multimodal semantic segmentation, shown in Figure 1. The first approach involves merging multiple modalities of visual perception data and feeding them into a feature extraction module; the second approach involves separate feature extraction modules for the different modalities, which share the same structure; and the third approach involves optimizing feature extraction modules specifically for different modalities, with each modality utilizing a distinct feature extraction module. While the first method is the simplest, its effectiveness is often not optimal. The third method is challenging to implement as it requires the construction of the specialized feature extraction modules for each modality. Our approach improves upon the second method, enabling easy expansion to any modality without the need for modality-specific feature extraction module design.

Despite the above-mentioned significant progress in multimodal semantic segmentation, we have identified some remaining challenges. Firstly, simply adding modalities does not always improve accuracy and may, at times, result in decreased accuracy, contradicting the common belief that more modal information should lead to higher accuracy. Secondly, current multimodal semantic segmentation methods mostly rely on RGB images supplemented by another modality, lacking the capability to incorporate more modalities. Finally, previous multimodal semantic segmentation models require modification of the neural network backbone for specific sensors, making it a complex task for scalable multimodality, implying that optimizing the backbone is needed for each additional modality.

To address these issues, we propose a simple and easily expandable multimodal semantic segmentation model. The proposed model adopts the same feature extraction backbone to handle all modalities, facilitating the addition of modalities while keeping the model concise. We design two sets of branches: one as the main branch using RGB, and the other as an expandable branch incorporating additional modalities. To fully leverage the advantages of various modalities, we introduce the multimodal score module (MSM) to assess the importance of each additional modality feature in overall semantic segmentation. These additional modality features are incorporated into the RGB main branch via the features complementary module (FCM) to enhance the semantic segmentation performance. Additionally, we incorporate the residual idea to ensure that all modalities can better extract features at each stage.

## 2. Related Works

In this section, we will present the relevant research on semantic segmentation and multimodal semantic segmentation.

### 2.1. Semantic Segmentation

The Fully Convolution Network (FCN) [17], as the first deep learning model to achieve end-to-end training, achieved groundbreaking significance in the field of semantic segmentation. By utilizing deconvolution layers for upsampling, FCN cleverly transforms low-resolution feature maps into high-resolution segmentation results. The Unet [18] model, on the other hand, successfully integrates low-level and high-level feature information via unique skip connections, providing a classical solution for semantic segmentation.

The innovation of Pyramid Scene Parsing Network (PSPnet) [19] lies in pyramid pooling, a method that performs pooling operations on feature maps at multiple scales, thereby obtaining richer feature representations. Deeplabv3 [20], combining skip connections and dilated convolutions, further enhances the accuracy of semantic segmentation. The HRNet [21] model achieves precise segmentation at high resolutions by employing parallel convolution paths at multiple resolutions.

Recently, methods based on boundary retrieval have made significant progress in the field of semantic segmentation. Approaches like those presented in [22,23] transform semantic segmentation into a boundary retrieval problem, significantly improving the segmentation performance by learning boundary information, offering a new perspective for semantic segmentation research.

With the emergence of Vision Transformers (ViT) [24] in the field of computer vision, algorithms based on them have also demonstrated remarkable performance in semantic segmentation. Models like segFormer [25] and SEgementation TRansformer (SETR) [26] employ encoder structures similar to ViT and combine them with pixel-level segmentation methods, achieving efficient semantic segmentation. Swin-Unet [27] is a pure Transformer model designed specifically for medical image segmentation, where its encoder–decoder structure and skip connections ensure effective extraction and fusion of contextual features.

Despite the significant achievements of the aforementioned methods in semantic segmentation tasks related to visual perception in autonomous driving, pure RGB images often fail to provide sufficient information in practical applications. Additionally, RGB cameras may be affected by lighting conditions or exhibit image blur during rapid movements, posing challenges to the accuracy of semantic segmentation. Therefore, future research needs to explore further the fusion of multimodal data and the integration of other sensor technologies to enhance the robustness and accuracy of semantic segmentation.

### 2.2. Multimodal Semantic Segmentation

Research in multimodal semantic segmentation aims to compensate for the limitations of RGB images in information acquisition by integrating data from different types of sensors. The primary goal of this technology is to leverage the complementarity among different modalities to improve the accuracy and the robustness of semantic segmentation.

In the task of semantic segmentation with RGB-D data, where RGB data primarily focuses on color information and depth data that provides spatial information, the Gated-Residual Block [28] effectively combines these two types of information using a gating mechanism to exploit their complementarity. On the other hand, CANet [29] achieves interaction and fusion of RGB and depth information via an innovative co-attention module.

For RGB-T (RGB-Thermal) data, models such as RTFNet [30] and FuseSeg [31] utilize RGB-thermal fusion networks to combine RGB and thermal data, thereby improving the accuracy of semantic segmentation in urban scenes. This approach demonstrates the effectiveness of thermal imaging data in semantic segmentation tasks.

Additionally, research explores the combination of optical flow with RGB data to enhance the semantic segmentation performance in autonomous driving [32]. In the case of RGB-LiDAR, researchers propose various methods to fuse data from these two modalities, including perception-aware multisensor fusion methods and 2D prior-assisted laser point cloud semantic segmentation methods [33].

CMX [14] and TokenSelect [15], through the use of Transformers architecture, achieve cross-modal feature fusion, enhancing the accuracy of RGB-X semantic segmentation. This method processes features from different modalities in parallel and utilizes cross-modal interactions to generate more robust cross-modal feature representations. CMNeXt [16] achieves performance improvement in semantic segmentation by extending the model asymmetrically.

In general, the design of multimodal semantic segmentation models mainly involves two approaches: firstly, fusing inputs from multiple modalities as the input to the overall model. However, this method has significant limitations as it can only be designed for a specific modality. The second approach involves extracting features separately for each modality, requiring the use of different backbones for different types of the modality feature extraction tasks. Although this method demonstrates reasonable effectiveness in cross-modal semantic segmentation, designing complex feature extraction modules for the specific modalities makes it challenging to easily extend the modality types.

## 3. Methodology

We will elaborate the comprehensive architecture of the proposed model in this section, elucidating the pivotal modules and techniques employed for standardizing diverse modal transformations.

### 3.1. Framework Overview

**Figure 2:**
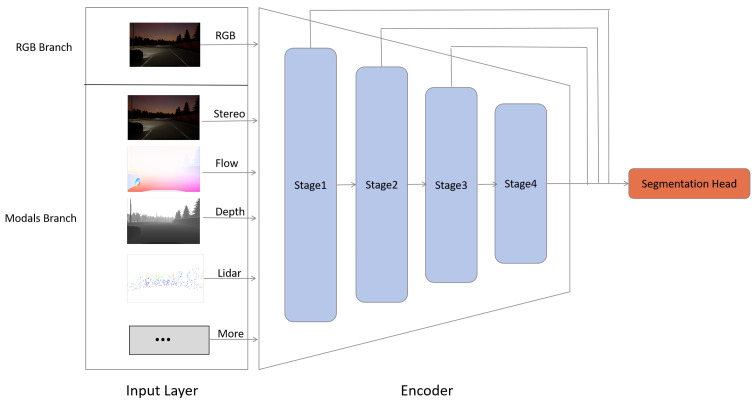
The Structure of Scalable Multimodal Semantic Segmentation Framework.

The proposed structure for our scalable multimodal semantic segmentation is based on an encoder–decoder architecture, as shown in Figure 2. Our model is logically designed with two branches: the main branch for RGB image feature extraction, and the secondary branch for extended modality feature extraction. Notably, the feature extraction part of our extended modality uses the exact same backbone. This approach makes it easier to handle additional modalities without the need to modify the backbone for each specific modality. In the field of computer vision, image processing models based on transformers, such as Vision Transformer (ViT) [24] and Swin Transformer (Swin) [34], continue to emerge. Through extensive prior research, it has been established that having four stages in the encoder yields optimal results. Consequently, to extract pyramid features, we have opted for a structure comprising four stages [35].

**Figure 3:**
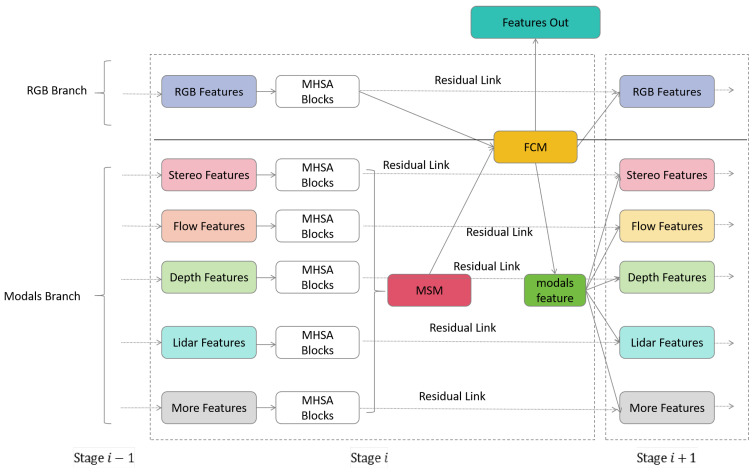
The Details of the Stage in Scalable Multimodal Semantic Segmentation Model.

We will illustrate the detailed encoder structure using stage *i* as an example i∈{0,1,2,3,4} , as shown in Figure 3. Here, stage 0 corresponds to the input layer, while the other stages share the same structure. Stage *i* consists of two branches: one is the RGB main branch, and the other is the auxiliary branch for extended modalities. Both branches derive their features from stage i−1 . The main branch RGB extraction incorporates the multi-head self-attention (MHSA) module [36], while each of the *M* extended modalities employs a block with the same structure to extract features.

This process yields M+1 features, denoted as $f_{RGB}$ and $f_{X_{m}}$ for *m*∈ $\{modal_{1},modal_{2}$ , ..., $modal_{N}\}$ . The *M* extended modality features $f_{X_{m}}$ go through the MSM module to obtain the fused extended modality feature $f_{X}$ . Then, $f_{RGB}$ and $f_{X}$ pass through the FCM module to produce the feature output $f_{out_{i}}$ for stage *i*, where i∈{1,2,3,4} . The next stage receives $f_{RGB}^{\prime }$ and $f_{X}^{\prime }$ as input, with $f_{X}^{\prime }$ being segmented back into its original modality form $f_{X_{m}}^{\prime }$ . We also incorporate the residual idea, retaining the previous $f_{RGB}$ and $f_{X_{m}}$ . In summary, the outputs $f_{RGB}+f_{RGB}^{\prime }$ and $f_{X_{m}}+f_{X_{m}}^{\prime }$ serve as inputs for the next stage.

**Figure 4:**
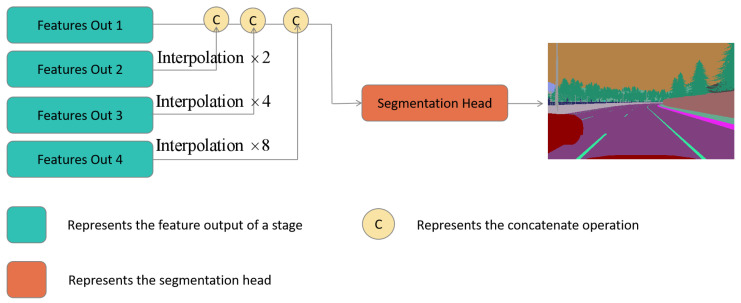
The Structure of Multimodal Semantic Segmentation Head.

After the four stages of the feature extraction, we obtain feature outputs $f_{out_{i}}$ for i∈{1,2,3,4} . These outputs are then input into the segment head to obtain the semantic segmentation image, as shown in Figure 4.

### 3.2. MSM and FCM

**Figure 5:**
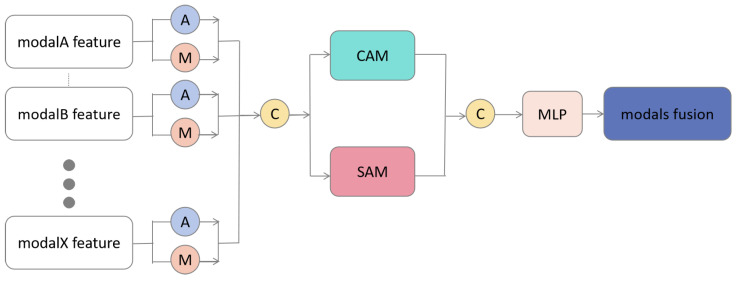
The Structure of Multimodal Score Module (MSM).

**Figure 6:**
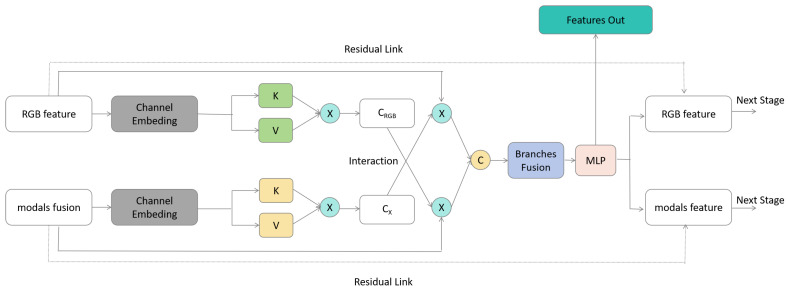
The Structure of Feature Complementary Module (FCM).

The Multimodal Score Module (MSM) and Feature Complementary Module (FCM) are pivotal components in constructing the scalable multimodal semantic segmentation model, as illustrated in Figure 5 and Figure 6, respectively.

In the MSM module, we perform max-pooling and average-pooling operations on the features of each extended modality $f_{X_{m}}$ for $m\in \{modal_{1},modal_{2}\cdots ,modal_{N}\}$ , obtaining two features that are subsequently merged into a fused feature $f_{X}$ . The fused feature then undergoes operations in the Channel Attention Module [37] and Spatial Attention Module [38]. The channel attention mechanism method is specified in Equation (1), and the spatial attention mechanism method is defined in Equation (2), of which these processes yields channel scores and spatial scores, respectively, for the extended modality.

(1) $$\begin{array}{c} W_{CAM}=Sigmoid\left(MLP\left(x\right)\right) \end{array}$$

(2) $$\begin{array}{c} W_{SAM}=\frac{exp(x_{i,j})}{\sum_{i,j}exp\left(x_{i,j}\right)} \end{array}$$

To compute channel attention, we feed the input feature *x* into a Multi-Layer Perceptron. After processing through the MLP , the resulting output is passed through a Sigmoid activation function to generate the channel attention weight, denoted as $W_{CAM}$ . In the calculation of the spatial attention mechanism, we compute attention weights for all feature pixels. Herein, *i* and *j* denote the indices of feature locations, and $x_{i,j}$ represents the feature value at that specific location. We utilize the exponential function $exp(x_{i,j})$ to determine the feature mapping values and obtain the spatial attention weight $W_{SAM}$ .

Through channel scores and spatial scores, we assess the channel importance and the spatial importance of the extended modality features. This operation is crucial, as features from different modalities contribute differently to the final semantic segmentation results. The merged feature then undergoes Multilayer Perceptron by Equation (3) to obtain a new fused feature $f_{X}$ , as defined in Equation (4),

(3) $$\begin{array}{c} x^{\prime }=MLP\left(x\right)=Sigmoid\left(Linear\left(ReLU\left(Linear\left(x\right)\right)\right)\right) \end{array}$$

where *x* is the input feature that undergoes processing through a linear layer Linear , followed by a non-linear transformation using the ReLU activation function. Subsequently, it passes through another linear layer Linear and is transformed by the Sigmoid activation function to yield the final output feature $x^{\prime }$ .

(4) $$\begin{array}{cc} \; & f_{CAM}=f+f\times W_{CAM}\\ \; & f_{SAM}=f+f\times W_{SAM}\\ \; & f_{X}=MLP\left(Concat\left(f_{CAM},f_{SAM}\right)\right) \end{array}$$

In this process, *f* serves as the input feature. Firstly, it is transformed by the channel attention weight $W_{CAM}$ to obtain the channel attention output feature $f_{CAM}$ . Subsequently, it is processed through the spatial attention weight $W_{SAM}$ to yield the spatial attention output feature $f_{SAM}$ . The features $f_{CAM}$ and $f_{SAM}$ are then combined using concatenation Concat . The concatenated feature undergoes further processing through a Multi-Layer Perceptron MLP before delivering the final output feature $f_{X}$ of the MSM module.

In the FCM, we ingeniously design a Cross Attention Mechanism, a transformation based on the traditional attention mechanism [36]. The input to the FCM module includes the main branch RGB feature $f_{RGB}$ and the secondary branch extended modality feature $f_{X}$ . We need to facilitate information interaction between these two branches. By using linear embedding for both the branches and then retaining the initial features $f_{X}$ and $f_{RGB}$ for the subsequent residual connections, we prepare for the later residual operations. Through the attention mechanism, we compute the Context Vectors $C_{RGB}$ and $C_{X}$ for both branches, as specified in Equation (5). The attention results are obtained by multiplying the Query and Context Vectors,

(5) $$\begin{array}{cc} \; & C_{RGB}=Softmax\left(K_{RGB}^{T}V_{RGB}\right)\\ \; & C_{X}=Softmax\left(K_{X}^{T}V_{X}\right) \end{array}$$

where $K_{RGB}^{T}$ and $K_{X}^{T}$ denote the keys for the RGB feature branch and the extended modality branch, respectively, while $V_{RGB}$ and $V_{X}$ represent the corresponding values for these branches. By applying the Softmax activation function, we determine the cross-attention weights, denoted as $C_{RGB}$ and $C_{X}$ .

We transform the attention mechanism into a Cross Attention Mechanism by exchanging information between the two branches, as defined in Equation (6). Subsequently, the features from both branches are merged and passed through MLP to obtain a stage feature output $f_{out}$ as specified in Equation (7). Finally, we separate the features back into the original two branches and connect them using the residual approach,

(6) $$\begin{array}{cc} \; & f_{RGB}^{\prime }=f_{RGB}+f_{RGB}C_{X}\\ \; & f_{X}^{\prime }=f_{X}+f_{X}C_{RGB} \end{array}$$

where $C_{X}$ denotes the attention weight for the extended modality branch, and $f_{RGB}$ represents the feature of the RGB branch. By employing the cross-attention mechanism, we obtain a new feature for the RGB branch, denoted as $f_{RGB}^{\prime }$ . Similarly, $C_{RGB}$ signifies the attention weight for the RGB branch, while $f_{X}$ stands for the feature of the extended modality branch. Through the utilization of the cross-attention mechanism, we can generate a new feature for the extended modality branch, denoted as $f_{X}^{\prime }$ ,

(7) $$\begin{array}{cc} \; & f_{out}=MLP\left(Concat\left(f_{RGB}^{\prime },f_{X}^{\prime }\right)\right) \end{array}$$

by performing concatenation Concat on multiple features and subsequently processing them through a Multi-Layer Perceptron MLP , we can obtain the feature output of a specific stage, denoted as $f_{out}$ .

### 3.3. Modal Data Representation

In the field of autonomous driving, leveraging multimodal data is crucial for enhancing the perceptual capabilities of the system. Commonly used data types include RGB images, depth images, flow maps, LiDAR data, infrared data, and polarization data. Each of these carries unique information contributing to a more comprehensive understanding of the driving environment. The transformation methods for these data types are detailed below.

RGB images represent the most common visual perception modality, where the information from three channels (red, green, and blue) simulate human visual perception. However, RGB images may face issues of overexposure or underexposure in excessively bright or dark scenes, affecting the accurate perception of the autonomous driving system. To address this, we normalize the range of RGB image data from [0, 255] to [0, 1] to better align with the input requirements of the semantic segmentation model.

Depth images provide information about the spatial depth of a scene, compensating for the lack of spatial depth information in RGB images. By transforming depth information into a standardized format, we can incorporate more texture, disparity, position, and contour information into the model. We convert the depth images into the HHA format [39], which provides geometric characteristics such as horizontal disparity, ground height, and angle information.

Flow maps contain information about the direction and the speed of motion for each pixel, exhibiting temporal characteristics and being unaffected by motion-induced blurriness. We convert the flow maps into a format that the model can process. Convert the u,v format of flow data into the R,G,B format of an image as defined in Equation (8), and finally, the R,G,B values need to be normalized,

(8) $$\begin{array}{cc} \; & map_{u}=\frac{flow_{u}}{max(\sqrt{\sum flow_{u}^{2}})}\\ \; & map_{v}=\frac{flow_{v}}{max(\sqrt{\sum flow_{v}^{2}})}\\ \; & R=1+map_{u}\\ \; & G=1-0.5(map_{u}+map_{v})\\ \; & B=1+map_{v} \end{array}$$

where $flow_{u}$ represents the motion vector in the *X*-axis direction of the image, while $flow_{v}$ represents the motion vector in the *Y*-axis direction. The magnitude of $flow_{u}$ and $flow_{v}$ indicates the amount of offset, and the positive or negative sign of $flow_{u}$ and $flow_{v}$ indicates the direction of the offset, respectively. $map_{u}$ and $map_{v}$ are intermediate mapping features for converting flow images to RGB images, respectively. We can colorize the optical flow calculation results using a color model, with $map_{u}$ and $map_{v}$ being the mapping process values for converting optical flow data to RGB data, respectively. We represent optical flow data in RGB form, ultimately yielding values for the R,G,B components.

LiDAR–camera fusion provides reliable and accurate spatial depth information about the physical world. To align the representation of LiDAR data with RGB images, we convert it into a format similar to a range-view image. The Field-of-View of the camera FV and the image size is H×W . The origin is (u0,v0)=(H/2,W/2) . Equation (9) defines the focal length $f_{x}$ and $f_{y}$ , respectively. Then, we use LiDAR 3D point cloud data to project it onto a 2D image by Equation (10),

(9) $$\begin{array}{cc} \; & f_{x}=H/\left(2tan\left(FV\times \pi /360\right)\right)\\ \; & f_{y}=W/\left(2tan\left(FV\times \pi /360\right)\right) \end{array}$$

where *H* and *W* are the 2D image’s height and width, respectively; FV is the Field-of-View of the camera; and $f_{x}$ and $f_{y}$ are intermediate features for 3D radar data to convert to 2D images. In practical calculations, we use 3.1415 as an approximation for π ,

(10) $$\begin{array}{c} \left[\begin{array}{c} u\\ v\\ 1 \end{array}\right]=\left[\begin{array}{cccc} f_{x} & 0 & u_{0} & 0\\ 0 & f_{y} & v_{0} & 0\\ 0 & 0 & 1 & 0 \end{array}\right]\left[\begin{array}{cc} R & t\\ 0 & 1 \end{array}\right]\left[\begin{array}{c} X\\ Y\\ Z\\ 1 \end{array}\right] \end{array}$$

where X,Y,Z are the points of the LiDAR data; u,v are the 2D image pixels; *R* are the rotation matrices; and *t* are the translation matrices [40].

Near-infrared light consists of electromagnetic waves of different wavelengths. The radiation behavior of light varies with the wavelength, and objects exhibit different colors because their reflectivity depends on the wavelength. To better integrate infrared data with other modalities, we conduct necessary preprocessing and transformation.

Polarization data is a specific type in the field of autonomous driving [41], capturing information about the polarization state of reflected light to perceive details such as road signs, lane markings, and traffic signals. This allows the autonomous driving system to more accurately identify and interpret road signs and other targets. We apply specific transformation methods to convert polarization data into a format which can be handled by the model. In Equation (11), the image consists of four aligned pixel images obtained at polarization angles of $I_{0{}^{\circ}},I_{45{}^{\circ}},I_{90{}^{\circ}},I_{135{}^{\circ}}$ , and $S_{0}$ represents the total light intensity, while $S_{1}$ and $S_{2}$ represent the ratios of linear polarization at 0° and 45° to their respective perpendicular polarized components. The polarization state of light, represented by $S_{0}$ , $S_{1}$ , and $S_{2}$ , can be derived from $I_{0{}^{\circ}},I_{45{}^{\circ}},I_{90{}^{\circ}},I_{135{}^{\circ}}$ ,

(11) $$\begin{array}{cc} \; & S_{0}=I_{0{}^{\circ}}+I_{90{}^{\circ}}=I_{45{}^{\circ}}+I_{135{}^{\circ}}\\ \; & S_{1}=I_{0{}^{\circ}}-I_{90{}^{\circ}}\\ \; & S_{2}=I_{45{}^{\circ}}+I_{135{}^{\circ}} \end{array}$$

in Equation (11), we use two representations: Degree of Linear Polarization (DoLP) and Angle of Linear Polarization (AoLP), as specified in Equation (12).

(12) $$\begin{array}{cc} \; & f_{DoLP}=\frac{\sqrt{S_{1}^{2}+S_{2}^{2}}}{S_{0}}\\ \; & f_{AoLP}=\frac{1}{2}\arctan\frac{S_{1}}{S_{2}} \end{array}$$

where $f_{DoLP}$ and $f_{AoLP}$ represent the transformed features, specifically the Degree of Linear Polarization and Angle of Linear Polarization characteristics, respectively.

Through these transformation methods, we standardize multimodal data into a format the model can process. This simplifies the model and significantly enhances the performance of semantic segmentation by synthesizing information from various modalities.

## 4. Experiments

This section will outline the datasets employed in our model experimentation and will also elaborate on the experimental parameters and showcase the experimental outcomes alongside their analysis.

### 4.1. Dataset

To validate the effectiveness of the model proposed in this paper, we conducted experiments using the SHIFT dataset [42] and the MCubeS dataset [43]. The SHIFT dataset is a comprehensive simulated autonomous driving dataset that offers rich sensor data, including RGB images, stereo images, depth images, optical flow maps, and LiDAR data. Generated in a highly realistic manner, these data comprehensively simulate various scenarios in real driving environments, providing invaluable support for research in autonomous driving technology. The images in the SHIFT dataset have a resolution of 1280×800 , comprising 150,000 training images and 25,000 validation images. The dataset encompasses 23 different semantic classes, offering abundant materials for our experiments in multimodal semantic segmentation.

Additionally, we chose the MCubeS dataset for experimentation. The MCubeS dataset incorporates various modalities, such as RGB, Near-Infrared (NIR), Degree of Linear Polarization (DoLP), and Angle of Linear Polarization (AoLP), focusing on semantic material segmentation for 20 classes. The images in this dataset have a resolution of 1224 × 1024, consisting of 302 training images and 90 validation images. A notable feature of this dataset is the presence of paired multimodal data, making it highly suitable for validating the effectiveness of our multimodal fusion and feature complementation methods. By conducting experiments using these two datasets, we can comprehensively assess the performance of our model in the context of autonomous driving scenarios.

### 4.2. Experiment Setup

To ensure the accuracy, consistency, and fairness of our experiments, we adopted a uniform set of parameter configurations throughout the entire research process. Specifically, we fixed the number of iterations at 300,000, utilized Stochastic Gradient Descent (SGD) as the optimizer, and set the batch size to 8. We employed the cross-entropy loss function. For more detailed model training hyperparameters, please refer to Table 1. We set the learning rate to 0.01 and utilized SGD as the optimizer. The Dropout rate is configured as 0.1, and the activation function chosen is ReLU. The width of the MHSA (Multi-Head Self-Attention) blocks is set to [64, 128, 320, 512], while the depth is set to [3, 4, 6, 3].

Regarding the experimental environments, we operated on the Windows 11 operation system and implemented them on the PyTorch 1.6 deep learning framework. Our hardware configuration included an INTEL Core i9-13900 processor (Santa Clara, CA, USA) and two NVIDIA GeForce RTX 3090 graphics cards (Santa Clara, CA, USA). To streamline the computational load, we downsized the resolution of all data to 640×400 .

### 4.3. Experiment Results

Through the extensive comparative experiments, including traditional RGB image semantic segmentation models such as Unet, PSPnet, Deeplabv3+, HRnet, and state-of-the-art multimodal models like TokenSelect and CMNeXt, we aimed to evaluate the impact of different modalities on the semantic segmentation performance. Using traditional RGB image semantic segmentation models as a baseline, we conducted further comparisons by incorporating various modalities into the models and contrasting their performance against state-of-the-art multimodal semantic segmentation models. Metrics such as mean accuracy and mean IoU are employed as benchmarks for model evaluation, with tests conducted on the SHIFT and MCubeS datasets.

As illustrated in Table 2, experiments were conducted on the SHIFT dataset, initially utilizing the performance of traditional RGB image semantic segmentation models as the baseline. We then explored the influence on model accuracy by progressively adding different modalities. A counterintuitive observation emerged from our experiments: the inclusion of stereoscopic imagery in the PSPnet, a traditional RGB image semantic segmentation model, resulted in lower accuracy compared to using only RGB images; the Deeplabv3+ model showed a similar situation. This discrepancy is attributed to the fact that traditional semantic segmentation models like PSPnet were not specifically optimized or calibrated for this modality. Additionally, we have conducted performance comparisons with the multimodal model MMAF-Net [44], specifically optimized for the RGB-depth modality. In the RGB-depth approach, methods specifically tailored for multimodal scenarios consistently outperform traditional approaches by a significant margin. Our model outperformed others when incorporating the stereoscopic visual modality. The addition of depth imagery further demonstrated the superiority of our model compared to other models.

Our proposed model is specifically optimized for extended modalities, particularly in the MSM (Modality-specific Self-attention) and FCM (Feature Calibration Module) modules. The MSM module employs a cross-attention mechanism, allowing the RGB main branch to acquire features from the extended modality, thereby improving the semantic segmentation performance. The FCM module combines spatial attention and channel attention, as the contribution of each modality may vary. Traditional semantic segmentation models do not undergo these optimizations, so sometimes adding modalities performs even worse than using only RGB for semantic segmentation. Both tokenselect and CMNeXt are also optimized for multimodal semantic segmentation, making them superior to traditional semantic segmentation models. Our proposed model even slightly outperforms the state-of-the-art multimodal semantic segmentation model CMNeXt.

**Figure 7:**
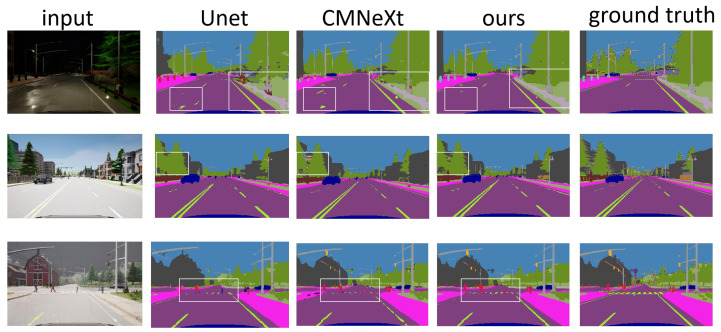
The Visualization of Experiment Results.

Expanding our experiments on the SHIFT dataset to include multiple modalities’ stereoscopic imagery, depth imagery, optical flow imagery, and radar imagery, our model consistently maintained its superiority over other advanced multimodal models. The performance improvement over traditional RGB semantic segmentation models was substantial, affirming the benefits of incorporating diverse modalities for semantic segmentation tasks. The visualization results can be seen in Figure 7, which demonstrates that the images segmented by our model are more accurate compared to other models.

Moreover, we conducted additional experiments on the MCubeS dataset, the experiment results are shown in Table 3, employing a similar baseline approach with traditional RGB image semantic segmentation models and assessing the impact of simple multimodal processing on these models. Consistent with the findings from the SHIFT dataset experiments, a decline in accuracy was observed when introducing certain modalities on the MCubeS dataset.

For instance, the incorporation of infrared imagery resulted in a reduction in accuracy for the PSPnet model compared to using RGB images alone. Similar accuracy declines were observed for both Deeplabv3+ and PSPnet with the addition of the DoLP (Degree of Linear Polarization) modality, underscoring the non-arbitrary nature of modality integration; thus, specific optimization for each modality is imperative.

Furthermore, as we introduced more modalities, including infrared imagery, the DoLP modality, and the AoLP (Angle of Linear Polarization) modality, our model consistently outperformed other state-of-the-art multimodal semantic segmentation models. This emphasizes the importance of tailoring the model’s architecture to the unique characteristics of each modality for optimal performance in multimodal scenarios. The results showcase the robustness and effectiveness of our proposed model in handling diverse modalities within the MCubeS dataset.

The notable improvement in performance can be attributed to the incorporation of the MSM and FCM modules into our model. These modules play a crucial role in enhancing the model’s ability to effectively process and integrate information from diverse modalities. Unlike traditional semantic segmentation models, our approach leverages the specific characteristics of each modality through the use of the MSM module, which employs a cross-attention mechanism. This enables the RGB main branch to capture features from the extended modalities, contributing to a significant boost in the semantic segmentation performance. Additionally, we included performance comparisons with some multimodal models optimized specifically for the RGB-thermal modality, such as FEANet [45]. In the RGB-thermal approach, methods tailored for multimodal scenarios consistently outperform traditional approaches by a significant margin.

In comparison to tokenselect and CMNeXt, our model outperforms these state-of-the-art multimodal semantic segmentation models. This superiority is particularly evident when handling the MCubeS dataset and incorporating various modalities, such as infrared imagery, DoLP modality, and AoLP modality. The comprehensive attention mechanisms implemented in our MSM and FCM modules, considering both spatial and channel attention, contribute to the model’s robustness in capturing distinctive features from different modalities.

We conducted ablation experiments on the SHIFT dataset and the MCubeS dataset by comparing the removal of the MSM module using a simple concatenate method (Concat) to merge features from the extended modal branch, the removal of the FCM module using a simple concatenate method (Concat) to merge features from the RGB main branch, and scenarios where residual connections were omitted. As shown in Table 4 and Table 5, the results indicate that removing each proposed module led to a decrease in model accuracy. This is because the MSM module employs a cross-attention mechanism, allowing the RGB main branch to better integrate features from the extended modal branch, which is more advantageous for overall performance improvement in semantic segmentation tasks compared to simple concatenate methods (Concat). Additionally, the FCM module, via the combination of the spatial attention mechanism and the channel attention mechanism, can extract more crucial features between different modalities, as the contribution of features between different modalities to the final semantic segmentation varies. Residual connections, inspired by the classical residual concept, ensure that the model maintains a certain level of robustness. These results confirm the beneficial impact of the introduced modules on multimodal semantic segmentation tasks.

## 5. Conclusions

In this paper, we propose a novel multimodal semantic segmentation model. By combining the multimodal data and the deep learning techniques, our model can perform semantic segmentation tasks more effectively. Specifically, we design multiple modules, including the multimodal fusion module (MSM) and the feature complementation module (FCM), to fully utilize the complementarity of information between different modalities. These modules can capture and fuse the features from the different modalities, thereby improving the accuracy of semantic segmentation.

To verify the effectiveness of our model, we conducted extensive experiments on the SHIFT and MCubeS datasets, which represent different challenges and complex scenarios in the field of autonomous driving. Compared with traditional semantic segmentation models and the advanced multimodal models, our method outperforms them in terms of average accuracy and Mean IoU. In particular, after adding the multiple extended modalities, our model even outperforms the latest multimodal models. These experimental results demonstrate the superior performance of our model in semantic segmentation tasks.

To further explore the specific contributions of each component in our model for performance, we conducted an ablation experiment. By removing the MSM module, the FCM module, and the residual connection one by one from the model, we found that the absence of any one component resulted in a decrease in performance, which fully demonstrates the important role of these components in our model and their contribution to the model’s performance improvement.

Although our model has achieved significant performance improvement in the experiments, there are still some challenges and future research directions. Firstly, we can further explore more effective multimodal fusion strategies to fully leverage the complementary information among different modalities. Secondly, we can investigate how to introduce semi-supervised learning methods into our model to reduce the dependence on large amounts of the labeled data. In addition, we can also consider applying our model to the other fields.

Finally, we look forward to further promoting the development of multimodal semantic segmentation technology via continuous research and optimization, making more beneficial contributions to the research and application of autonomous driving.

## Figures and Tables

**Table 1:** Model Training Hyperparameters.

Hyperparameter Names	Value
Batch Size	8
Learning Rate	0.01
Optimizer	SGD
%midrule Dropout Rate	0.1
Activation Function	ReLU
MHSA Block Width	[64, 128, 320, 512]
MHSA Block Depth	[3, 4, 6, 3]

**Table 2:** Experimental results on the SHIFT dataset.

	Modal	Mean Acc (%)	Mean IoU (%)
HRnet	RGB-only	45.5	39.1
PSPnet	RGB-only	39.8	33.8
Unet	RGB-only	44.3	38.5
Deeplabv3+	RGB-only	**47.4**	**41.0**
ours	RGB-only	43.3	37.2
PSPnet	RGB-Stereo	39.2	33.6
HRnet	RGB-Stereo	45.7	39.2
Unet	RGB-Stereo	45.3	39.3
Deeplabv3+	RGB-Stereo	41.8	35.4
tokenselect	RGB-Stereo	42.8	36.6
CMNeXt	RGB-Stereo	47.9	41.2
our	RGB-Stereo	**49.0**	**42.2**
PSPnet	RGB-Depth	45.7	39.9
Deeplabv3+	RGB-Depth	51.2	43.0
MMAF-NET	RGB-Depth	54.6	48.1
tokenselect	RGB-Depth	55.2	48.9
CMNeXt	RGB-Depth	54.3	47.9
our	RGB-Depth	**57.8**	**51.3**
tokenselect	RGB-S-D-F-L	55.1	48.9
CMNext	RGB-S-D-F-L	57.5	51.1
ours	RGB-S-D-F-L	**57.9**	**51.5**

The values in bold indicate the best performance in this set of experiments.

**Table 3:** Experimental results on the MCubeS dataset.

	Modal	Mean Acc (%)	Mean IoU (%)
PSPnet	RGB-only	**31.8**	**23.9**
HRnet	RGB-only	29.7	21.0
Deeplabv3+	RGB-only	29.9	21.2
ours	RGB-only	29.6	21.2
PSPnet	RGB-NIR_warped	30.7	22.5
Deeplabv3+	RGB-NIR_warped	30.4	21.3
FEANet	RGB-NIR_warped	32.1	23.0
tokenselect	RGB-NIR_warped	31.5	22.8
CMNeXt	RGB-NIR_warped	32.3	23.1
ours	RGB-NIR_warped	**32.9**	**23.5**
PSPnet	RGB-DoLP	29.7	22.5
Deeplabv3+	RGB-DoLP	29.8	21.0
tokenselect	RGB-DoLP	31.8	22.8
CMNeXt	RGB-DoLP	**32.2**	**23.1**
ours	RGB-DoLP	**32.2**	**23.1**
tokenselect	RGB-AoLP	32.9	23.3
CMNeXt	RGB-AoLP	33.1	23.5
ours	RGB-AoLP	**33.2**	**23.6**
tokenselect	RGB-N-D-A	35.6	26.0
CMNeXt	RGB-N-D-A	36.6	26.4
ours	RGB-N-D-A	**37.0**	**27.0**

The values in bold indicate the best performance in this set of experiments.

**Table 4:** Ablation experiments on the SHIFT dataset.

	Mean Acc (%)	Mean IoU (%)
full	**57.9**	**51.5**
MSM → Concat	55.1	48.9
FCM → Concat	56.7	50.3
-Residual	57.5	51.1

The values in bold indicate the best performance in this set of experiments.

**Table 5:** Ablation experiments on the MCubeS dataset.

	Mean Acc (%)	Mean IoU (%)
full	**37.0**	**27.0**
MSM → Concat	33.0	23.8
FCM → Concat	34.6	25.1
-Residual	34.3	24.9

The values in bold indicate the best performance in this set of experiments.

## Data Availability

The data presented in this study are available on request from the corresponding author.
